# Sensitivity Analysis in Nonrandomized Longitudinal Mediation Analysis

**DOI:** 10.3389/fpsyg.2021.755102

**Published:** 2021-12-06

**Authors:** Davood Tofighi

**Affiliations:** Department of Psychology, University of New Mexico, Albuquerque, NM, United States

**Keywords:** mediation analysis, sensitivity analysis, no omitted confounder assumption, latent growth analysis, structural equation model (SEM)

## Abstract

Mediation analysis relies on an untestable assumption of the no omitted confounders, which posits that an omitted variable that confounds the relationships between the antecedent, mediator, and outcome variables cannot exist. One common model in alcohol addiction studies is a nonrandomized latent growth curve mediation model (LGCMM), where the antecedent variable is not randomized, the two covarying mediators are latent intercept and slope modeling longitudinal effect of the repeated measures mediator, and an outcome variable that measures alcohol use. An important gap in the literature is lack of sensitivity analysis techniques to assess the effect of the violation of the no omitted confounder assumption in a nonrandomized LGCMM. We extend a sensitivity analysis technique, termed correlated augmented mediation sensitivity analysis (CAMSA), to a nonrandomized LGCMM. We address several unresolved issues in conducting CAMSA for the nonrandomized LGCMM and present: (a) analytical results showing how confounder correlations model a confounding bias, (b) algorithms to address admissible values for confounder correlations, (c) accessible R code within an SEM framework to conduct our proposed sensitivity analysis, and (d) an empirical example. We conclude that conducting sensitivity analysis to ascertain robustness of the mediation analysis is critical.

## Introduction

**FIGURE 1:**
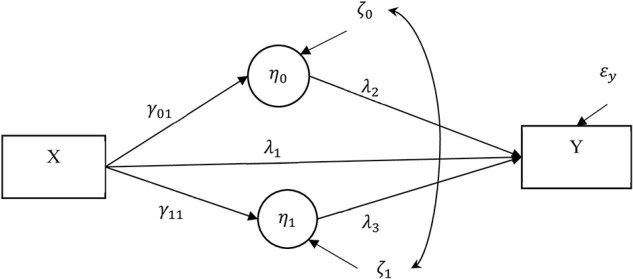
Latent growth curve mediation model (LGCMM). The antecedent variable (*X*) was pain at 4 months after treatment. The two covarying mediators were the latent intercept (η_0_ = mean negative mood at 4 months) and slope (η_1_ = monthly rate of negative affect) for the repeated measures of negative affect. The outcome variable (*Y*) measures percent drinking days (PDD) at 16 months. *C* denotes a set of covariates (e.g., background variables). A single-headed arrow shows the direct effect of a variable at the origin on the variable at the end of the arrow. A curved double-headed arrow shows covariance between the two residuals.

Mediation analysisC has become more common in analyzing complex causal chains in health and psychological studies. One common model in alcohol addiction studies (e.g., Moyers et al., 2009; Hartzler et al., 2011; Maisto et al., 2015) is a nonrandomized latent growth curve mediation model (LGCMM; von Soest and Hagtvet, 2011). The LGCMM, as shown in Figure 1, hypothesizes that a nonrandomized antecedent variable (pain) influences both mediators (i.e., mean negative affect and monthly rate of negative affect); these mediators, in turn, cause an outcome variable (alcohol use). Further, the antecedent variable can be also randomized (randomized mediation model). A critical, yet untestable assumption in any mediation model, including the LGCMM, is the no omitted confounder assumption (Judd and Kenny, 1981; Robins and Greenland, 1992; Pearl, 2014; MacKinnon and Pirlott, 2015; Valente et al., 2017). A no omitted confounder assumption states that an omitted variable (confounder) may not exist if it confounds the relationships among the antecedent, mediators, and outcome variable. In a randomized mediation model, this assumption implies that, by properly randomizing the antecedent variable, we can rule out the effect of a confounder on the antecedent variable to the mediators and on the outcome variable relationships but not on the mediators to outcome relationships. In a nonrandomized mediation model, however, an omitted confounder can influence *all* the relationships among antecedent variable, mediators, and outcome variable. As a result, it is more challenging to assess the impact of violating the no confounding assumption because the additional patterns of confounding can happen with a nonrandomized mediation model when that model is compared to a randomized mediation model. Because the no omitted confounder assumption is not testable and because the proper randomization of the antecedent and mediator variables is absent, researchers have recommended sensitivity analysis (Imai et al., 2010; VanderWeele and Arah, 2011; Tofighi et al., 2013, 2019; Albert and Wang, 2015; VanderWeele, 2015; Tofighi and Kelley, 2016). A sensitivity analysis assesses the impact of various degrees of violation of the no omitted confounder assumption on the model parameter estimates and on any inferences about the indirect effects.

Despite the prevalence of nonrandomized longitudinal mediation studies in areas such as alcohol addiction (e.g., Moyers et al., 2009; Hartzler et al., 2011; Maisto et al., 2015), most research attention has been on randomized mediation model in both multilevel/longitudinal and single-level data as a means of improving causal inference. In fact, to our knowledge, no study to date has offered a method to conduct sensitivity analysis for a nonrandomized longitudinal mediation model with two covarying mediators in a structural equation model (SEM), a multivariate framework to study covariance structure. Previous research mostly focused on the sensitivity analysis for a randomized model with a single-level data in SEM or in a potential outcome framework (Imai et al., 2010; Cox et al., 2013; Albert and Wang, 2015; Valente et al., 2017; Hong et al., 2018; Lindmark et al., 2018; McCandless and Somers, 2019). For a mediation model with two independent mediators, Imai and Yamamoto (2013) studied sensitivity analysis and strongly assumed independence between the two mediators; their technique, however, cannot be directly applied to a model with covarying mediators as it could result in bias in estimating indirect effects (VanderWeele, 2015). Several studies proposed randomized and nonrandomized sensitivity analysis for sequential mediation models, where one mediator is assumed to sequentially cause another mediator, in both randomized (Imai and Yamamoto, 2013; Daniel et al., 2015) and nonrandomized models (Harring et al., 2017). Because of the strong assumption that the mediators are measured in chronological order, the model specification, interpretation, and sensitivity analysis techniques developed for a sequential mediation model are not directly applicable to a model with covarying mediators, where the mediators freely covary but do not causally impact one another. In multilevel/longitudinal mediation analysis, methods to conduct sensitivity analysis have been proposed for nonrandomized (Tofighi and Kelley, 2016) and for randomized single-mediator model (Bind et al., 2016; Talloen et al., 2016). For two mediators, Tofighi et al. (2019) proposed an SEM-based sensitivity analysis method for a randomized LGCMM. However, Tofighi et al. (2019) did not consider a nonrandomized model where a confounder can influence a pair of relationships among antecedent variable, mediators, and outcome variable. To our knowledge, no study to date has extended sensitivity analysis to a nonrandomized LGCMM in an SEM framework.

Nonrandomized LGCMM poses interwoven challenges compared to either single-level or longitudinal randomized models. First, nonrandomization means a confounder can impact the antecedent as well as the mediators and the outcome variable. Thus, a confounder may impact not only the relationships between the mediators and the outcome variable (as in a randomized mediation model) but also may affect additional relationships of the antecedent to each mediator variable and to the outcome variable. This potential influence poses two additional challenges. The first issue is how to model and estimate biasing impact of a confounder on the antecedent variable in an SEM framework if the antecedent variable is exogenous. In a situation where an antecedent variable is exogenous, the covariates, if they exist, do not influence the antecedent variable. This is a critical issue to address in sensitivity analysis because, in an SEM framework for mediation analysis, an antecedent variable (randomized or not) is generally modeled as an exogenous (and fixed) rather than an endogenous (and random) variable when the covariates are not assumed to influence the antecedent variable. The challenge is to propose a method to convert the antecedent variable without a predicting covariate that is modeled as an exogenous variable into an endogenous variable so that potential impact of omitted confounder on the antecedent variable can be modeled through a confounder correlation.

The second challenge arises because of the additional relationships that can be influenced by a confounder in a nonrandomized model compared to a randomized model. In this instance, conceptualizing, estimating, and interpreting all the confounding relationships and their impacts on the indirect effect estimates will be more complicated than any other sensitivity analysis that has been performed. Also, in a longitudinal model, the repeated measures are correlated; thus, special techniques such as multilevel modeling (Raudenbush and Bryk, 2002) are required to account for lack of independence and to make correct inference about uncertainty of the parameter estimates. In addition, because of the multilevel structure of data, confounders can impact the model variables at different levels of aggregation (Tofighi and Kelley, 2016), and, thus, techniques developed for a single-level model may not be directly applicable. Further, the existence of two covarying mediators requires that the indirect effect through each mediator be simultaneously estimated. Conducting sensitivity analysis for each mediator separately while ignoring the other covarying mediators, as is done in a single-mediator model, is likely to result in bias because the two mediators are covarying (VanderWeele, 2015). Thus, techniques developed for a single-mediator model cannot be directly used to conduct sensitivity analysis in a two covarying mediator model. Lastly, given a variety of patterns of confounding bias, summarizing the impact of confounding bias succinctly enables researchers to assess sensitivity of the parameter estimate as well as statistical inference to the confounding bias. Given the importance of nonrandomized longitudinal model in practice and the unresolved practical and theoretical issues that hinder conducting sensitivity analysis for such models, proposing a method on how to conduct sensitivity analysis that can be implemented in SEM framework using available software packages is critical.

In this paper, we extend a sensitivity analysis technique from a randomized longitudinal mediation model to a complex, nonrandomized longitudinal mediation model, such as the model illustrated in Figure 1, in an SEM framework. More specifically, we extend a technique, termed *correlated augmented model sensitivity analysis* (CAMSA), that was developed for a randomized longitudinal mediation model to conduct sensitivity analysis in nonrandomized longitudinal mediation model with two covarying mediators (Tofighi et al., 2019). The extended CAMSA augments a nonrandomized mediation model with confounder correlations induced by a hypothesized confounder and addresses the unresolved challenges mentioned previously in modeling the biasing impact of the confounder bias. We present analytic results showing the confounder correlations are a function of omitted confounder relations to the model variables; we thereby show how the confounders correlations can be used to estimate confounding biases. We further present results on how to model and estimate confounder correlations in a nonrandomized longitudinal mediation model using the lavaan package (Rosseel, 2012), an opensource, freely available SEM package within the R statistical computing software framework (R Development Core Team, 2020). We present R code along with detailed instruction and an empirical example on how to conduct, interpret, and present the results of this proposed sensitivity analysis^1^.

## Sensitivity Analysis for Nonrandomized Mediation Model

In this section, we extend CAMSA to conduct sensitivity analysis for LGCMM (Figure 1). For simplicity but without loss of generality, we consider an LGCMM without any covariates. However, the results presented in this section will hold when adding the covariates to LGCMM in Figure 1 as we will demonstrate in the empirical example section. A crucial step in CAMSA is to specify a set of confounder correlations; a confounder correlation is used to model the impact of the omitted confounders on the model parameters. More specifically, confounder correlations are specified between the residuals associated with the endogenous variables (i.e., the variables with arrows pointed toward them). In extending CAMSA to the nonrandomized LGCMM, we faced several challenges. First, we needed to determine how to specify confounder correlations between an antecedent variable, which is an exogenous variable with no residual term, and the residuals associated with the endogenous variables in the model. This step is required because CAMSA uses confounder correlations to model biasing confounder effects. Second, we found a lack of clarity about whether the confounder correlations are uniquely a function of the confounder effects on the model parameters or whether they are also a function of the existing relationships between the variables. Clarifying such relationships would elucidate what confounder correlations are modeling. Thus, it is necessary to analytically demonstrate relationships between the confounder correlations and the effect of the confounder on the model parameters. Third, because of an infinite number of the combinations of confounder correlations, we were challenged to find plausible values for confounder correlations that are admissible and practical. In finding admissible values, we employ and implement different methods such that the correlation matrix is positive definite. To find sensible values, we propose steps to explore a finite set of plausible confounder correlation patterns as opposed to examining an infinite number of patterns; thus, we provide a more accessible way for researchers to conduct and interpret sensitivity analysis when facing an infinite number of choices for confounder correlations. In the next section, we introduce and extend CAMSA to the nonrandomized LGCMM. We then present formulae for computing confounder correlations in CAMSA. Next, we show equivalency between the correlated augmented model and a latent augmented model, an LGCMM that models a confounder explicitly. Finally, we present methods to generate admissible confounder correlations for CAMSA.

### CAMSA

**FIGURE 2:**
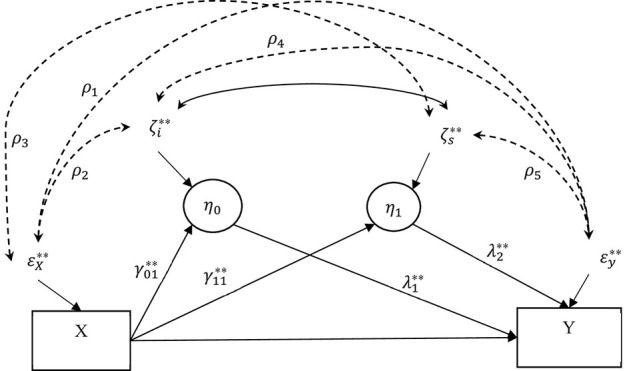
Correlated augmented LGCMM. The antecedent variable (*X*) was pain at 4 months after treatment. The two covarying mediators were the latent intercept (η_0_ = mean negative mood at 4 months) and slope (η_1_ = monthly rate of negative affect) for the repeated measures of negative affect. The outcome variable (*Y*) measures percent drinking days (PDD) at 16 months. A single-headed arrow shows the direct effect of a variable at the origin on the variable at the end of the arrow. A solid double-headed arrow shows covariance between the two residuals. A dashed doubled headed arrow shows confounder covariance (correlation) between the residuals.


To conduct CAMSA and address the challenges outlined above, we first specify the correlated augmented model, which adds to the original LGCMM (Figure 1) with the confounder correlations as shown in Figure 2. But first, we address the challenge that, in the model in Figure 1, the antecedent variable is not endogenous variable but is an exogenous variable without a residual term. To solve this issue, we introduce a residual term ε_*xi*_ for the antecedent variable *X*. To ensure that the model is identified, we fix variance of the residual term to equal the variance of the antecedent variable. The residual term would explicitly specify the antecedent variable as an endogenous variable as opposed a ‘‘fixed’’ exogenous variable that tends to be a default setting in SEM software packages. That is, specifying this residual term will convert the role of the antecedent variable from exogenous to endogenous, thus permitting us to specify the confounder correlations between the antecedent variable residual term and the other residual terms in the model. Now, we can model confounder bias as the confounder correlations among all the residual terms of the endogenous variables. In addition, by converting the antecedent variable to an endogenous variable, we can manipulate elements of the covariance matrix of endogenous variables. Below we present the equations for this model where Eq. (1) demonstrates specification for converting the antecedent variable into an endogenous variable. The superscript ‘‘^∗∗^’’ denotes the parameters for the correlated augmented model^2^.

(1) $$x_{i}=\alpha_{3}^{**}+\varepsilon_{x\,i}^{**}$$

(2) $$m_{i\,j}=\eta_{i\,0}+\eta_{i\,1}\,t_{i\,j}+e_{i\,j}^{**}$$

(3) $$\eta_{i\,0}=\alpha_{0}^{**}+\gamma_{01}^{**}\,x_{i}+\zeta_{0\,i}^{**}$$

(4) $$\eta_{i\,1}=\alpha_{1}^{**}+\gamma_{11}^{**}\,x_{i}+\zeta_{1\,i}^{**}$$

(5) $$y_{i}=\alpha_{2}^{**}+\lambda_{1}^{**}\,x_{i}+\lambda_{2}^{**}\,\eta_{i\,0}+\lambda_{3}^{**}\,\eta_{i\,1}+\varepsilon_{y\,i}^{**}$$

In the above equations, subscript *i* denotes person *i* = 1, …, *N*, and subscript *j* denotes an occasion for the repeated measures variable *m*_*ij*_ where *j* = 1,…, *p*. Variables *m*_*ij*_ and *t*_*ij*_ are the repeated measures on the observed mediators and the time score, respectively; *y_i* is the outcome variable, and *x_i* is the antecedent variable. Latent intercept and slope are denoted by η_*i*0_ and η_*i*1_. The terms $\alpha_{0}^{**}$ and $\alpha_{1}^{**}$ denote the intercepts for the latent growth factors. The intercept for the outcome variable is $\alpha_{2}^{**}$; the intercept for the antecedent variable is $\alpha_{3}^{**}$ where it is, in fact, estimated by the sample mean for the antecedent variable. The parameters $\gamma_{01}^{**}$ and $\gamma_{11}^{**}$ quantify the effects of the antecedent variable on the latent intercept and slope, respectively. The regression coefficients $\lambda_{1}^{**},\lambda_{2}^{**}$, and $\lambda_{3}^{**}$ quantify the effects of the antecedent variable, latent intercept, and slope on the outcome variable, respectively.

The second part of positing the correlated augmented model is to specify the variances and covariances between the residuals for the model. From a multilevel perspective, two levels of residuals for LGCMM exist. First, there are *p* Within (Level-2) residuals associated with repeated measures *m*_*ij*_s, $\varepsilon_{W}^{**}={\left(e_{i\,1}^{**},\ldots ,e_{i\,p}^{**}\right)}^{T}$, where T denotes vector transpose operator. Second, there are four Between (Level-2) residuals $\varepsilon_{B}^{**}={\left(\varepsilon_{i\,x}^{**},\varepsilon_{i\,y}^{**},\zeta_{0\,i}^{**},\zeta_{1\,i}^{**}\right)}^{T}$, where $\varepsilon_{i\,x}^{**},\varepsilon_{i\,y}^{**},\zeta_{0\,i}^{**},\text{and}\,\zeta_{1\,i}^{**}$ are associated with the antecedent variable, outcome variable, and latent intercept and slope, respectively. Note that $\varepsilon_{i\,x}^{**}$ is included in vector of the residuals because we explicitly specify the antecedent variable as an endogenous variable with a residual term. We assume that the covariances between the Level-1 and Level-2 residuals to be zero (Raudenbush and Bryk, 2002).

For each level, the vector of residuals has the multivariate normal distribution with a mean vector of zero and a covariance matrix. For the Within residuals, the upper-triangle covariance matrix is:


$$\Sigma_{W}^{**}=\left[\begin{array}{ccc} \sigma_{e_{1}^{**}}^{2} & 0 & 0\\ & \ddots & 0\\ & & \sigma_{e_{p}^{**}}^{2} \end{array}\right]$$

where $\sigma_{e_{1}^{**}}^{2}$ and $\sigma_{e_{p}^{**}}^{2}$ are the residual variance for *m*_*i1*_ and *m*_*ip*_, respectively. We assume that the covariances between the repeated measures are zero although one could estimate the Within residuals when this premise is supported by theory. For the Between residuals, the upper-triangle covariance matrix is:

$$\Sigma_{B}^{**}=\left[\begin{array}{cccc} \sigma_{\varepsilon_{x\,i}^{**}}^{2} & \sigma_{\varepsilon_{x\,i}^{**}}\,\sigma_{\varepsilon_{y\,i}^{**}}\,\rho_{1} & \sigma_{\varepsilon_{x\,i}^{**}}\,\sigma_{\zeta_{0}^{**}}\,\rho_{2} & \sigma_{\varepsilon_{x\,i}^{**}}\,\sigma_{\zeta_{1}^{**}}\,\rho_{3}\\ & \sigma_{\varepsilon_{y\,i}^{**}}^{2} & \sigma_{\varepsilon_{y\,i}^{**}}\,\sigma_{\zeta_{0}^{**}}\,\rho_{4} & \sigma_{\varepsilon_{y\,i}^{**}}\,\sigma_{\zeta_{1}^{**}}\,\rho_{5}\\ & & \sigma_{\zeta_{0}^{**}}^{2} & \sigma_{\zeta_{0}^{**},\zeta_{1}^{**}}\,\rho_{\eta_{0},\eta_{1}}\\ & & & \sigma_{\zeta_{1}^{**}}^{2} \end{array}\right].$$

As shown in Figure 2, ρ_1_, ρ_2_, and ρ_3_ are the confounder correlations between the antecedent variable and outcome variable, the latent intercept and slope, respectively; ρ_4_ and ρ_5_ are the confounder correlations between the latent intercept and slope and the latent slope and outcome variable, respectively. The terms σ^2^s denote the variances of the respective residuals.

The confounder correlations ρ_1_ to ρ_5_ are assumed to model the effect of the omitted confounder bias on the model parameters although the exact nature of the relationships between the confounder correlations and the omitted confounder remains unclear. Note that if we had assumed that all the confounders were included in the model (e.g., the covariates included the confounders), most if not all the confounder correlations would equal zero (Tofighi et al., 2013, 2019; Tofighi and Kelley, 2016). If all the confounders were included in the model, except for the residuals of the latent intercept and slope, the residuals associated with the antecedent variable, latent intercept and slopes, and the outcome variable would not correlate merely because of the omitted confounders^3^. This argument will be made clearer when we show the relationships between the confounder correlations and the omitted confounder effects later in this article. The covariance of the residuals between the latent intercept and slope is usually freely estimated (Singer and Willett, 2003). In general, the covariance between the intercept and slope should not be fixed at zero because of its potential substantive interpretation (Snijders and Bosker, 2011). As we will discuss later, the covariance between the intercept and slope could be biased because of the confounder bias when *ρ*s are non-zero.

Before conducting CAMSA using the correlated augmented model, three important issues must be addressed. First, we need to derive analytical formulas to transform the confounder correlations into confounder covariances that are covariances between the residuals quantifying the effects of the omitted confounders. Second, we need to determine the relationships between the confounder correlations and the effect of the omitted confounder on the model parameters. Third, we need methods to generate admissible confounder correlation values that are of substantive interest. We address these issues in the next sections.

### Transforming Confounder Correlations Into Confounder Covariances

In conducting CAMSA, we cannot use confounder correlations directly to specify a correlated augmented mediation model in SEM framework because of scaling of the endogenous variables. Rather, we need to use the confounder covariance and estimates of the residual variance and then convert the fixed values of confounder correlations to confounder covariance using the derived computational formulas. We use the derived formulas to estimate the correlated augmented model (see Supplementary Material for details on deriving the formulas).


$$c\,o\,v\,\left(x_{i},\sigma_{\varepsilon_{y\,i}^{**}}\right)=\rho_{1}\,\sigma_{\varepsilon_{x\,i}^{**}}\,\sigma_{\varepsilon_{y\,i}^{**}}$$

$$c\,o\,v\,\left(x_{i},\zeta_{0\,i}^{**}\right)=\rho_{2}\,\sigma_{\varepsilon_{x\,i}^{**}}\,\sigma_{\zeta_{0\,i}^{**}}$$

$$c\,o\,v\,\left(x_{i},\zeta_{1\,i}^{**}\right)=\rho_{3}\,\sigma_{\varepsilon_{x\,i}^{**}}\,\sigma_{\zeta_{1\,i}^{**}}$$

$$c\,o\,v\,\left(\zeta_{0\,i}^{**},\varepsilon_{y\,i}^{**}\right)=\rho_{4}\,\sigma_{\zeta_{0\,i}^{**}}\,\sigma_{\varepsilon_{y\,i}^{**}}$$

$$c\,o\,v\,\left(\zeta_{1\,i}^{**},\varepsilon_{y\,i}^{**}\right)=\rho_{5}\,\sigma_{\zeta_{1\,i}^{**}}\,\sigma_{\varepsilon_{y\,i}^{**}}$$

### Equivalence Between Correlated Augmented Model and Latent Augmented Model

**FIGURE 3:**
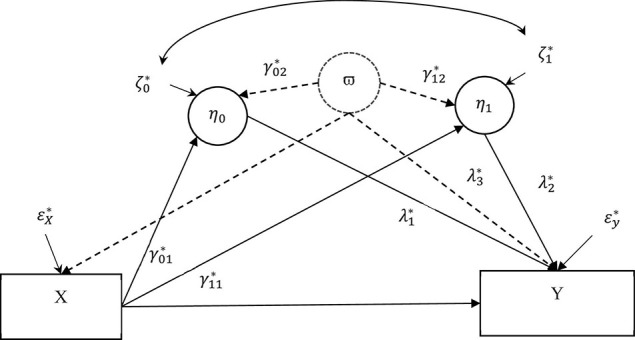
A latent augmented LGCMM. The antecedent variable (*X*) was pain at 4 months after treatment. The two covarying mediators were the latent intercept (η_0_ = mean negative mood at 4 months) and slope (η_1_ = monthly rate of negative affect) for the repeated measures of negative affect. The outcome variable (*Y*) measures percent drinking days (PDD) at 16 months. A solid single-headed arrow shows the direct effect of a variable at the origin on the variable at the end of the arrow. A solid double-headed arrow shows covariance between the two residuals. The dashed circle shows the latent proxy variable *ϖ* and the dashed arrows show the confounder parameters modeling the effect of the latent proxy variable on the endogenous variables.

In this section, we show the equivalence between the correlated augmented model used in CAMSA and the *latent augmented model* in Figure 3. We use the term *latent augmented model* because *ϖ*, termed a *latent confounder*, denotes a latent variable that accounts for a linear combination of the potential omitted confounders. Establishing equivalency is critical because it is unclear whether the confounder covariances/correlations in the correlated augmented model exclusively account for the confounder correlations or for correlations not caused by an omitted confounder. Model specification and equations for the latent augmented model and detailed analytic proofs of the equivalency between the latent augmented model and correlated augmented model are shown in Supplementary Material.

A significant contribution of our paper is to establish that the latent augmented model is equivalent to the correlated augmented model. That is, there is a one-to-one relationship between the corresponding parameters from the two model. To confirm this correspondence, we must show that the confounder correlations/covariances in the correlated augmented model are, in fact, functions of the confounding parameters in the latent augmented model. It is not trivial that the confounder correlations/covariances specifically model the effects of confounders and not the other relationships between the variables in the model. For example, we show that the covariance between the latent intercept and slope in the correlated augmented model is not, in general, equal to the corresponding covariance between the intercept and the slope in the latent augmented model.

Using a latent augmented model in sensitivity analysis is an extension of the sensitivity analysis technique used in randomized LGCMM (Tofighi et al., 2019) and multilevel SEM (Tofighi and Kelley, 2016) and is similar to the phantom variable technique used in single-level SEM (Harring et al., 2017). We only use the latent augmented model to exhibit equivalency but not to conduct sensitivity analysis because using the latent augmented model over the correlated augmented model potentially produces negative residual variance (Tofighi et al., 2019). Furthermore, using a latent augmented model means that the confounder parameters, regression coefficients measuring confounding bias on the model variables, are not easily interpretable because of scaling of the variables. These issues are remedied in the correlated augmented model because we use confounder correlations, which are effect size measures, and thus are more easily interpreted to gauge the impact of confounding bias.

### Generating Confounder Correlation Matrix

In the correlated augmented model, the confounder correlations are a set of fixed values that researchers establish with some restrictions that will make correlation values admissible. We will discuss different methods of finding admissible confounder correlation values. One important contribution of our model is that we propose a two-step procedure to investigate values for the confounder correlations that has not been used in sensitivity analysis literature. The two-step procedure uses the following two methods of generating admissible correlation values: (a) Toeplitz matrix method and (b) nearest positive-definite (PD) matrix method. We explain in detail how each method works and enumerate the pros and cons of each method. We show application of the two-step procedure using an empirical example in the next section.

A critical issue that needs to be addressed before conducting CAMSA is finding admissible values for the confounder correlations. Finding correlation confounder values is challenging because the correlations have a restricted range of [−1, 1] and are restricted by the values of other confounder correlations. In other words, the correlation values are not independent. This dependency means that we cannot pick values for a correlation independent of the values of other correlations. For example, for a triplet of correlations, all the values must satisfy the following constraint (Rousseeuw and Molenberghs, 1994):

$$\rho_{12}^{2}+\rho_{13}^{2}+\rho_{23}^{2}-2\,\rho_{12}\,\rho_{13}\,\rho_{23}\leq 1$$

Finding the values of the triplets of correlations that would satisfy the above restriction is not straightforward, especially as the number of correlations increase. This challenge can be more readily seen when arranging correlation values into a matrix as the Between confounder correlation matrix shown below.


(6) $$\left[\begin{array}{cccc} 1 & \rho_{1} & \rho_{2} & \rho_{3}\\ & 1 & \rho_{4} & \rho_{5}\\ & & 1 & \rho_{\eta_{0},\eta_{1}}\\ & & & 1 \end{array}\right]$$

The correlation matrix in (6) must be positive semi-definite (PSD). A square symmetric matrix is PSD if and only if the matrix determinant is greater than or equal to zero. Finding the determinant and setting it to be greater than (equal to) zero would provide us with necessary and sufficient condition for the correlation matrix. However, generating a matrix that would satisfy these conditions is not straightforward. Additionally challenging is generating a symmetric PSD whose diagonal elements are one and off-diagonal elements are between −1 and 1 where the correlation values substantively meaningful. The challenges are to find values and patterns of confounder correlations that are of substantive interest while satisfying the PSD condition. Below we discuss a two-step procedure for generating PSD correlation matrices.

#### Step 1: Toeplitz Matrix Method

One way to generate a correlation matrix is to use a special type of symmetric Toeplitz matrix (Schott, 1997; Wicklin, 2015) in which the main diagonal and diagonals parallel to the main diagonal are constant. We focus on the symmetric Toeplitz matrix whose diagonal elements are one. Consider *n* real numbers *a*_0_,*a*_1_,…,*a*_*n*−1_where *a*_0_=1. Then we can denote a symmetric Toeplitz matrix whose first row is *a*_0_,*a*_1_,…,*a*_*n*−1_ by


(7) $$T_{n}=T_{n}\,\left[a_{0},a_{1},\ldots ,a_{n-1}\right]=\left[\begin{array}{cccc} a_{0} & a_{1} & \cdots & a_{n-1}\\ & a_{0} & \cdots & a_{n-2}\\ & & \ddots & \vdots \\ & & & a_{0} \end{array}\right]$$

We use the results by Bogoya et al. (2012) to generate a special case for Toeplitz matrix where the matrix is square and symmetric with the main diagonal of one. A main result of Bogoya et al. (2012) is that a symmetric Toeplitz matrix whose first row is a linearly decreasing sequence (i.e., a sequence that decreases by the same amount each time) of non-negative values is PD. Thus, the process creates a sequence of decreasing positive values that generates a correlation matrix (Wicklin, 2015). To illustrate, consider a general polynomial sequence of the form *c*_1_−(*j*−1)*c*_2_ where *c_1* and *c_2* are constant and *j* is the index for column number, *j* = 1,…,*n*, — although one could also build a Toeplitz matrix with the first column and then use a row index. The sequence is expanded as follows: *c*_1_,*c*_1_−*c*_2_,*c*_1_−2*c*_2_,…. A necessary condition for the matrix to be PD is that the sequence should positive, thus *c*_1_−(*j*−1)*c*_2_ > 0. For a correlation matrix, we set *c*_1_=1 because the main diagonal elements of a correlation matrix equal one. Thus, the condition 1−(*j*−1)*c*_2_ > 0 means that decrement *c_2* must satisfy *c*_2_ < 1/(*n*−1) and that the largest value for *j* is the dimension of the matrix, *n*. For example, a decrement for a triplet correlation Toeplitz matrix must be *c*_2_ < 1/2. For the triplet of correlation values, when *c*_2_=1/4, the first row is (1,3/4,1/2), and the resulting correlation matrix is


$$\left[\begin{array}{ccc} 1 & 3/4 & 1/2\\ & 1 & 3/4\\ & & 1 \end{array}\right].$$

To generate this matrix in R, we use


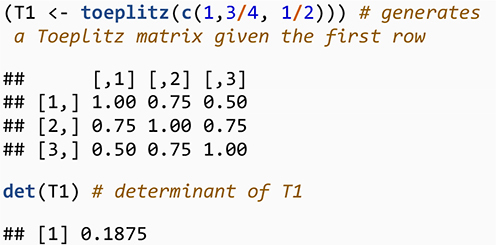


What about the Toeplitz matrix for 4×4 confounder correlation matrix for our LGCMM? The first row for the Toeplitz for the confounder correlation matrix is (1,1−*c*_2_,1−2×*c*_2_,1−3×*c*_2_) where *c*_2_ < 1/3. We wrote an R function to generate the first row and used Toeplitz function in R to generate the Toeplitz confounder correlation matrix.


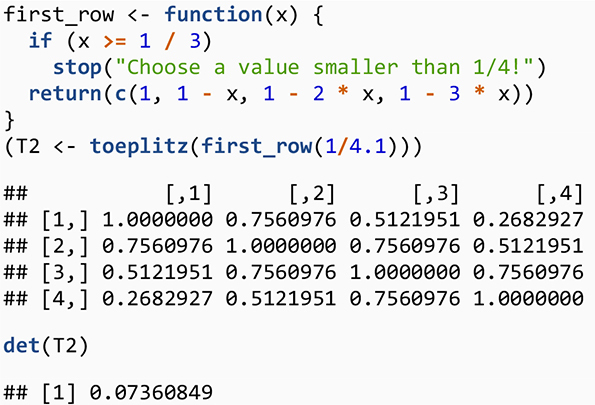


So far, we have talked about generating the confounder correlation matrix using Toeplitz matrix and a result from Bogoya et al. (2012) that only generates positive confounder correlations. What if we would like to have negative confounder correlations as well? Bogoya et al. (2012) extended their results to include the negative correlation by showing that a linearly decreasing sequence of numbers can include negative values if the sum of the values in the sequence remains positive, that is,


$$\sum_{j=1}^{n}c_{1}-\left(j-1\right)\,c_{2}=n\,c_{1}-c_{2}\,\sum_{j=1}^{n}\left(j-1\right)$$

$$\;\;=nc_{1}-c_{2}\frac{n\,\left(n-1\right)}{2}>0.$$

Given the *c*_1_=1 for a Toeplitz correlation matrix, we have


$$n-c_{2}\,\frac{n\,\left(n-1\right)}{2}>0$$

$$2-c_{2}\,\left(n-1\right)>0$$

$$c_{2}<\frac{2}{\left(n-1\right)}$$

The above result indicates that, if we want to create a Toeplitz correlation matrix with both positive and negative confounder correlations, then we must choose a decrement that satisfies the condition $c_{2}<\frac{2}{\left(n-1\right)}$. However, if we want to generate a correlation matrix with positive values, the decrement must satisfy this condition $c_{2}<\frac{1}{\left(n-1\right)}$. Note that the decrement for the positive confounder correlation is smaller than one for positive and negative confounder correlation. Now, we modify our R function to indicate that one may choose a decrement that would generate a Toeplitz matrix that includes both positive and negative correlation matrix:


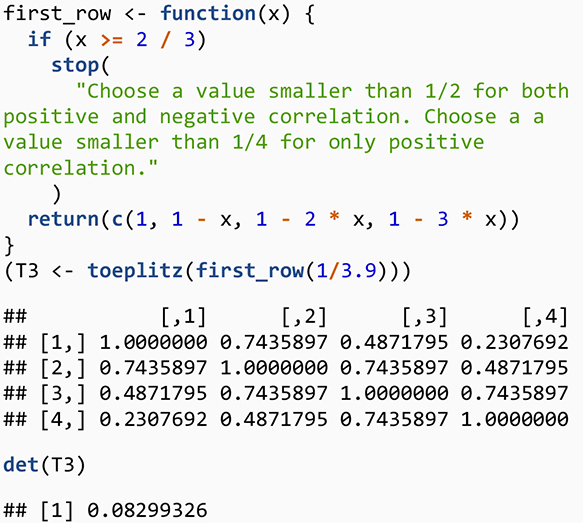


Using this algorithm provides a great flexibility in choosing a select number of confounder correlations to examine a relatively wide range of the indirect effect values as well as to examine model convergence. Thus, we recommend this algorithm be used as an initial step to inspect the correlation confounders and indirect effects values as well as the non-convergence of the mediation model. While relative simplicity of this method and the relationship between the confounder correlations dictated by Toeplitz algorithm are advantages, the algorithm also limits the range of confounder values because the confounder correlation values follow the Toeplitz algorithm. Thus, we recommend researchers use this initial step to investigate confounder correlation values and their impacts on the sensitivity of the indirect effects as well as on the model convergence.

#### Step 2: Nearest Positive-Definite Method

In the second step, we examine in more depth the range of confounder correlation values that led to convergence of the model using the relevant information from Step 1. Examining the range of possible values would exhaust the memory and computational resources. Even a more limited range of confounder correlations could take hours on faster available PCs. Given that we will generate thousands of correlation matrices, using the confounder correlation values from the initial phase will help us focus on the select ranges of the confounder correlations and, thus, be able to examine more thoroughly the combination of confounder correlation values within the select ranges gleaned from Step 1.

In this second step, we use the range of values that do not lead to non-convergence of the model to generate many correlation matrices to be used in sensitivity analysis. However, as mentioned before, not all combinations of the correlation values would lead to PD confounder correlation matrices. To solve this problem, we use an algorithm suggested by Higham (2002) to transform a non-PD correlation matrix into a “nearest” PD matrix. The nearest PD matrix is achieved by repeatedly projecting the original non-PD matrix onto the set of all symmetric positive semidefinite matrices (termed *cone*) with unit diagonal entries.

The benefit of Higham’s (2002) algorithm is that we can choose and control the range of values for each confounder correlation. For example, we can choose a few values for ρ_*XY*_, such as small, medium, and large according to Cohen’s (1988) guideline, while we choose a continuous range for other confounder correlations, for example, 0≤ρ_*XM*1_≤0.5. A limitation of this method is that the many combinations of the confounder correlation values can lead to non-convergence of the model. As a result, this method can be computationally expensive even with the computational power of the modern computers. Further, this method is a compromise between having more control over the range of the confounder correlation values and the convergence rate of the model. Next, we illustrate an application of our proposed CAMSA to an empirical example and show that CAMSA is generalizable to a nonrandomized LGCMM with covariates.

## Empirical Example

To illustrate the application of CAMSA to a nonrandomized longitudinal growth model, we used data from Combined Pharmacotherapies and Behavioral Interventions for Alcohol Dependence study (COMBINE; The COMBINE Study Research Group, 2003), a randomized control trial that studied 16 weeks of active treatment alcohol use disorder on 1,383 participants recruited across 11 sites. The participants received nine individual treatments or a combination of the following treatments: sobriety and enhance medication adherence training (Medical Management, MM), individualized psychotherapy for outpatient alcohol dependence (combined behavioral intervention, CBI), medications to reduce alcohol dependency (e.g., acamprosate, naltrexone, or combination of the two), or a placebo. Background information (covariates) prior to treatment and assessment measures at the beginning (baseline).

In our example, we were interested in whether the negative effect of pain on a participant’s drinking outcome would be mediated through negative affect. The antecedent variable was pain at 4 months (the end of the treatment). Pain was measured by two items. One item, selected from the 26-item World Health Quality of Life assessment (World Health Organization, 1997), asks “To what extent do you feel that physical pain prevents you from doing what you need to do?” The possible responses range from 1 “not at all” to 5 an “extreme amount.” The second question, selected from the 12-item Short Form Health Survey (Ware et al., 1996), asks “During the past 4 weeks, how much did pain interfere with your normal work including both work outside the home and housework?” Again, the possible responses range 1 “not at all” to 5 “extremely.” The outcome variable was percent drinking days (PDD) at 16 months and was measured via Form 90 (Miller, 1996).

The two mediators were the intercept and slope for repeated measures of negative affect. Negative affect was measured by the self-reported, 53-item Brief Symptom Inventory (BSI) that measures distress (Derogatis and Melisaratos, 1983). An example item asks, “How much were you distressed by nervousness or shakiness inside?” with responses ranging from 0 “not at all” to 4 “extremely.” The BSI was measured at 4 months (the end of treatment), 6.5, 13, and 17 months. For the LGCMM, the latent intercept measured the mean negative affect at 4 months while the latent slope measured the monthly rate of negative affect.

For this example, we also controlled for the following covariates measured at or prior to the baseline: demographics (i.e., gender, marital status, employment status, income, and minority status), baseline alcohol dependence severity (Skinner and Allen, 1982), number of alcohol dependence symptoms (American Psychiatric Association, 1995), readiness to change (DiClemente and Hughes, 1990), and alcohol abstinence self-efficacy (DiClemente et al., 1994). Our proposed CAMSA results are generalizable to LGCMM with the covariates. That is, the analytical results for confounder correlation conversion formulas and equivalency still hold. One notable adaptation when adding covariates is that, because we control for the covariates for antecedent variable as well as the mediators and outcome variable, the antecedent variable is automatically endogenous. Thus, we did not need to explicitly specify the antecedent variable as an endogenous variable.

We fitted LGCMM using lavaan (Rosseel, 2012) and conducted CAMSA in R (R Development Core Team, 2020), an open source, freely available statistical software^4^. If the no-omitted-confounder assumptions hold, the indirect effect through the intercept was 0.096 (*SE* = 0.014), 95% CI [0.07, 0.125] and the indirect effect through the slope was −0.013 (*SE* = 0.012), 95% CI [−0.039, 0.011]. Recall that the latent intercept was the mean negative mood at 4 months and latent slope was the monthly rate of negative affect. These results indicate that pain increased the mean of negative mood at 4 months and that, in turn, increased PDD at 16 months. However, pain does not appear indirectly to change PDD through the negative mood monthly change.

Given that the no-omitted confounder assumption is not testable, we used our proposed method to conduct sensitivity analysis. An important step was to find correlation confounder values that were both feasible and practical. To do that, we followed the steps of our proposed method. In Step 1, we generated structured Toeplitz correlation matrices using the algorithm by Bogoya et al. (2012). We then augmented the model with confounder correlation values, ran the model, and computed the indirect effect estimates for each model. To save space, we only show a few select combinations of confounder correlations and the indirect effect through latent intercept and slope in Tables 1, 2, respectively; more complete tables containing the results can be found in the Supplementary Materials. We found that not all combinations of confounder correlation values would result in convergence. Confounder correlation values equal or greater than the values ρ_*XY*_≥−0.032, ρ_*XM*_1__≥0.656, ρ_*XM*_2__≥0.312, ρ_*M*_1_*M*_2__≥0.656, ρ_*M*_1_*Y*_≥0.312, ρ_*M*_2_*Y*_≥0.656 resulted in nonconvergence. Nonconvergence means that the confounder correlation values caused model nonconvergence, and, thus, these confounder correlations were inadmissible. Although these values were proper confounder correlation values from the standpoint of the confounder correlation matrix being PD, the values were not compatible with the data and the model implied correlation structure; thus, these values were discarded. If we fix a correlation, or any parameter for that matter, that is not supported by the data and the model, then the chance of model nonconvergence increases (Anderson and Gerbing, 1988).

**TABLE 1:** A sample of sensitivity analysis results for indirect effect through intercept for zero to small confounder correlation.

**ρ_*XY*_**	**ρ_*XM*_1__**	**ρ_*XM*_2__**	**ρ_*M*_1_*M*_2__**	**ρ_*M*_1_*Y*_**	**ρ_*M*_2_*Y*_**	**Indirect effect**	**LL**	**UL**
**Non-significant indirect effects**								
−0.1	0.1	0	−0.1	0.1	0	0.012	−0.00005	0.02447
−0.1	0.1	0	−0.05	0.1	0	0.012	−0.00005	0.02447
−0.1	0.1	0	0	0.1	0	0.012	−0.00005	0.02447
−0.1	0.1	0	0.05	0.1	0	0.012	−0.00005	0.02447
−0.1	0.1	0	0.1	0.1	0	0.012	−0.00005	0.02447
**Largest indirect effects**								
−0.1	−0.1	0	−0.1	−0.1	0	0.249	0.203	0.295
−0.1	−0.1	0	−0.05	−0.1	0	0.249	0.203	0.295
−0.1	−0.1	0	0	−0.1	0	0.249	0.203	0.295
−0.1	−0.1	0	0.05	−0.1	0	0.249	0.203	0.295
−0.1	−0.1	0	0.1	−0.1	0	0.249	0.203	0.295

*These results are from Step 1, where the structured Toeplitz correlations, *ρ*s, were generated using the algorithm by Bogoya et al. (2012). LL, lower limit; UL, upper limit.*

**TABLE 2:** A sample of sensitivity results for largest and smallest indirect effect through slope for zero to small confounder correlations.

**ρ_*XY*_**	**ρ_*XM*_1__**	**ρ_*XM*_2__**	**ρ_*M*_1_*M*_2__**	**ρ_*M*_1_*Y*_**	**ρ_*M*_2_*Y*_**	**Indirect effect**	**LL**	**UL**
**Smallest indirect effect**								
0.1	0.1	0	−0.1	−0.1	0	−0.0167	−0.0487	0.0153
0.1	0.1	0	−0.05	−0.1	0	−0.0167	−0.0487	0.0153
0.1	0.1	0	0	−0.1	0	−0.0167	−0.0487	0.0153
0.1	0.1	0	0.05	−0.1	0	−0.0167	−0.0487	0.0153
0.1	0.1	0	0.1	−0.1	0	−0.0167	−0.0487	0.0153
**Largest indirect effect**								
−0.1	0.1	0	−0.1	0.1	0	−0.0086	−0.0255	0.0082
−0.1	0.1	0	−0.05	0.1	0	−0.0086	−0.0255	0.0082
−0.1	0.1	0	0	0.1	0	−0.0086	−0.0255	0.0082
−0.1	0.1	0	0.05	0.1	0	−0.0086	−0.0255	0.0082
−0.1	0.1	0	0.1	0.1	0	−0.0086	−0.0255	0.0082

*These results are from Step 1, where the structured Toeplitz correlations, *ρ*s, were generated using the algorithm by Bogoya et al. (2012). LL, lower limit; UL, upper limit.*

In Step 2, using the results from Step 1 as a guide, we explored a wider range of confounder correlation values. We used the near PD method, which allowed us to examine more combinations of confounder correlations for the sensitivity analysis. In addition, given that the range of confounder correlation values were not guaranteed to be PD, we used the near PD algorithm to convert a non-PD confounder correlation matrix into its nearest PD matrix. Like Step 1, we then augmented the model with confounder correlation values, ran the model, and computed the two indirect effect estimates for each model. The five confounder correlations took on five values, −0.3, −0.1, 0, 0.1, and 0.3, and resulted in 15,625 combinations. Of 15,625 estimates for each indirect effect, 15,000 (96%) resulted in nonconvergence.

Because of a relatively large number of estimates, we recommend researchers be deliberate in examining indirect effects for the corresponding range of confounder correlation values. We started by examining the results for zero to small effect (0 < ρ < 0.1) for the confounder correlations. For the indirect effect through the intercept, examining the range of values for small to zero showed support for the indirect effect to be robust in that indirect effect remained positive; further, the CI limits were all positive except for a few cases shown in Table 1. Maximum indirect effects when the confounder correlations were in the zero to small effect range are also shown in Table 1. For the medium to large confounder correlations, however, none of the models converged. Nonconvergence results should be interpreted in the context of the select values for the confounder correlations; we could not conclude that all the medium to large confounder correlations would result in nonconvergence.

For the indirect effect through slope when the confounder correlations were zero to small effect range, the sign of the magnitude and inference about the indirect effect CI limits remained unchanged. Table 2 shows five combinations of the confounder correlations that resulted in the smallest and the largest indirect effects. The indirect effect estimates remained negative, ranging from −0.0167 to −0.0086. The lower limit of the CIs ranged from −0.0487 to −0.0255 while the upper limit ranged from 0.0153 to 0.0082. Because the indirect effect CI contained zero, the indirect effect did not appear to be different from zero when the confounder correlations ranged from zero to small. Finally, we conducted sensitivity analysis when the confounder correlations ranged from medium to large with the values ranging from 0.3 to 0.5 and from −0.5 to −0.3. All the combinations resulted in nonconvergence.

One important feature of our proposed CAMSA is that we can ascertain sensitivity of the model fit to the confounder correlations by examining convergence of the model fit to the data. The nonconvergence results indicate that the correlated augmented model, which consists of the constraints imposed by the confounder correlations along with the implied covariance matrix and mean structure posited by the model, was not supported by the sample data (Anderson and Gerbing, 1988). The estimation algorithm was not able to find the sample estimates that would maximize the likelihood of data given the correlated augmented model. We concluded that the fit of the posited model itself was sensitive to select medium to large values of confounder correlations because the fit of the model was not supported by the sample data. One interpretation of model convergence sensitivity is that the effect of the confounder correlations would severely degrade the fit of the posited model to the sample data to a degree that the model would not be able to be estimated from the sample data. The fit of the posited model appeared to be sensitive to the confounders with medium to large influence on the model. Thus, we could argue that the posited model as a whole (global fit) and the indirect effects (local fit) as a part of the posited model do not appear to be robust to the confounder correlations ranging from medium to large effects.

In summary, it appears that when the confounder correlations were in the zero to small range, the overall model convergence and the two indirect effects through intercept and slope were less sensitive. For many of the combinations of the confounder correlations, the indirect effect results for the correlated augmented model remained the same as the ones for the posited model when the no omitted confounder was assumed. However, for the combinations of confounder correlations in medium to large range, the model showed high sensitivity that resulted in an overall lack of model convergence. As a result, we were not able to estimate the indirect effects for medium to large confounder correlations.

## Conclusion

A critical, yet untestable assumption in mediation analysis is the no omitted confounder assumption. This assumption states that an omitted confounder should not influence any pair of variables in a mediation model. Even when the antecedent variable (*X*) is randomized, one cannot rule out the effect of a confounder on the relationship between the mediator and outcome variable because the values of mediator (*M*) are not randomized. A more complicated situation is when the antecedent variable is not randomized and when we have two covarying mediators. For this model, a confounder could affect any pair of variables including the antecedent variable. Because the no omitted confounder assumption is untestable, researchers recommend conducting sensitivity analysis that ascertain the impact of potential confounders on the estimates and the possible inference about indirect effects (VanderWeele and Arah, 2011; Tofighi et al., 2013, 2019; Tofighi and Kelley, 2016; Valente et al., 2017). In this manuscript, we extend sensitivity analysis to a nonrandomized latent growth curve mediation model (LGCMM) with two covarying mediators in SEM framework. Conducting sensitivity analysis for the nonrandomized LGCMM has not been done before because certain challenges have interfered. First, nonrandomization means a confounder can impact the antecedent as well as the mediators and the outcome variable. A confounder may impact not only the relationships between the mediators and the outcome variable (as in a randomized mediation model) but also may affect additional relationships of the antecedent to each mediator variable and to the outcome variable. Second, a longitudinal model requires a more sophisticated statistical technique such as LGCMM that can address dependency in repeated measures while modeling mediation through two latent variables: latent intercept and slope. In LGCMM, when the is no covariate or the covariates do not affect the antecedent variable, the antecedent variable is exogenous. The issue remains on how to model and estimate biasing impact of a confounder on the exogenous antecedent variable in LGCMM. Further, the existence of two covarying mediators requires that the indirect effect through each mediator be simultaneously estimated. Conducting sensitivity analysis for each mediator separately while ignoring the other covarying mediators, as is done in a single-mediator model, is likely to result in bias because the two mediators are covarying (VanderWeele, 2015). Thus, techniques developed for a single-mediator model cannot be directly used to conduct sensitivity analysis in a two covarying mediator model. Lastly, given a variety of patterns of confounding bias, summarizing the impact of confounding bias succinctly enables researchers to assess sensitivity of the parameter estimate as well as statistical inference to the confounding bias.

We extended the sensitivity analysis technique termed CAMSA to a nonrandomized LGCMM. A major contribution of our method is the extension of sensitivity analysis to a nonrandomized antecedent variable. This expansion is significant because nonrandomized studies are common and pose additional challenges such as having to address more confounder relationships between the variables because of not having a randomized antecedent variable. Another contribution of our model is that we analytically showed that CAMSA is statistically equivalent to a model with an augmented latent confounder. The analytic work is important because we show how confounder correlations in CAMSA are directly a function of confounder effects in the equivalent latent augmented model. Without explicitly showing these relationships, what confounder correlation is modeling in CAMSA is unclear. Another advantage of our proposed method is that it is performed in SEM framework. The SEM framework allows researchers to, first, simultaneously estimate indirect effects through covarying mediators. Estimating indirect effects independently using separate regression equations would result in biased estimates of indirect effects. Second, researchers could check the effect of confounder correlations on model (global) fit and convergence of correlated augmented model in addition to modeling confounder effects on (local fit of) indirect effects. Examining convergence of the mediation model is a strength of using SEM to conduct mediation analysis and CAMSA because of the simultaneous estimation of multiple regression equations allows researchers to examine the convergence and the fit of mediation models to the sample data. If a specific range of confounder correlations could result in nonconvergence, then checking confounder effect on indirect effect would not be feasible. Third, as shown in the empirical example, existing SEM software can be used to conduct our proposed CAMSA. We provided code in R that would facilitate researchers in conducting the proposed CAMSA in their own research.

We recommend that researchers conduct sensitivity analysis and report the results to assess the robustness of mediation analysis to untestable assumption of no omitted confounders. Because the researchers in social science often use SEM to conduct a mediation analysis, our proposed method along with provided code for CAMSA in SEM context should be an attractive tool that would help researchers enhance robustness of their findings. In addition, we recommend researchers report, in a meaningful way, the range of values for which the results for indirect effects change; that is, investigators should describe when the inference about indirect effect changes. Finally, given the strength of SEM in assessing model fit and convergence of a model, we recommend researchers report ranges of confounder correlations that would result in nonconvergence. We further encourage researchers to explore the reasons for nonconvergence. One reason for nonconvergence is that the correlated augmented model is either inaccurately specified or too constrained to be supported by the sample data. That is, the specific range of confounder correlations render a model not supported by the data. For example, if zero to small range of confounder correlation would cause a large percentage of the nonconvergence, then one might conclude that the posited mediation model is itself sensitive and not robust to small changes. That conclusion would call into question the correct specification of the posited model and could motivate researchers to examine and modify the model carefully. If the model convergence is sensitive to medium and large range of confounder correlations, then researchers could reexamine specification of the posited mediation model. If the convergence rate would not improve, then the researchers could conclude that the model is robust to small range of confounder correlation but not to the medium and large values. The implication of each conclusion should be interpreted in the context of substantive research.

Limitations of the current research are that *X*, *M*, and *Y* are all continuous variables and that we, therefore, assume all the relationships are linear. Future research should extend these methods to a mediation model with one or more categorical outcome. Categorical outcome would require using generalized linear mixed model, and, thus, definition of indirect effects in the potential outcome framework should be used (VanderWeele, 2015). A further limitation of the current study is that we assumed that all variables in the model are measured without errors. While the latent intercept and slope can model measurement error, we did not use latent variables for *X* and *Y*. Future research should investigate the joint effects of confounder bias and measurement error (Fritz et al., 2016).

In sum, it is critical to conduct sensitivity analysis to ascertain robustness of the mediation analysis and carefully explain mediation analysis results in the context of correlation confounders and substantive research. Our proposed sensitivity analysis provides a tool for researchers to conduct sensitivity analysis for a nonrandomized LGCMM using available SEM software.

## Data Availability Statement

The data analyzed in this study is subject to the following licenses/restrictions: Access to COMBINE data files is restricted to the persons with a signed data access agreement from NIAAA. Requests to access these datasets should be directed to Raye Litten of NIAAA, rlitten@mail.nih.gov.

## Author Contributions

DT contributed to conception, design, and analysis of the study.

## Conflict of Interest

The author declares that the research was conducted in the absence of any commercial or financial relationships that could be construed as a potential conflict of interest.

## Publisher’s Note

All claims expressed in this article are solely those of the authors and do not necessarily represent those of their affiliated organizations, or those of the publisher, the editors and the reviewers. Any product that may be evaluated in this article, or claim that may be made by its manufacturer, is not guaranteed or endorsed by the publisher.

## Footnotes

1 The R script for our proposed CAMSA for the empirical example is provided in the online Supplementary Material.

2 Note that for simplicity and without loss of generality, we consider an LGCMM without any covariates. However, the results presented in this section hold when adding the covariates to LGCMM Figure 1 as will be shown in the “Empirical Example” section.

3 The residuals could be correlated due to other factors, such as common methods factors (Podsakoff et al., 2003).

4 The R script for our proposed CAMSA for the empirical example is provided in the online Supplementary Material.
