# Supplementary material for: Sensitivity Analysis in Nonrandomized Longitudinal Mediation Analysis
Source: Front Psychol. 2021 Dec 6;12:755102. doi: 10.3389/fpsyg.2021.755102 (PMC8685264; doi:10.3389/fpsyg.2021.755102)
Supplement: Supplementary file 1 [file Data_Sheet_1.docx]

# Supplemental Materials

We show equivalency between the latent augmented model the correlated augmented model. Our proposed latent augmented LGCMM is shown in Figure 3. The effects of the latent confounder *ϖ* on the endogenous variables are termed *confounder parameters*. Without loss of generality and for identification purposes, we assume that the latent proxy variable has the standard normal distribution (Tofighi et al., 2019; Tofighi & Kelley, 2016). Next, we derive analytic results to determine the relationships between the confounder parameters and confounder correlations/covariances and to measure the degree of the confounding bias. That is, we compute the relationships between the confounder parameters in the latent augmented model and the confounder correlations/covariances in the correlated augmented model.

We modify Equations 1- 5 to specify the latent augmented model in Figure 3. We use superscript “*” to denote the model parameters as follows.

$x_{i}=\alpha_{3}^{*}+\lambda_{5}^{*} \varpi+ \varepsilon_{xi}^{*}$ ()

$m_{ij}=\eta_{i0}+\eta_{i1} t_{ij}+ e_{ij}^{*}$ ()

$\eta_{i0}=\alpha_{0}^{*}+\gamma_{01}^{*} x_{i}+\gamma_{02}^{*} \varpi+\zeta_{0i}^{*}$ ()

$\eta_{i1}=\alpha_{1}^{*}+\gamma_{11}^{*} x_{i}+\gamma_{12}^{*} \varpi+ \zeta_{1i}^{*}$ ()

$y_{i}=\alpha_{2}^{*}+\lambda_{1}^{*} x_{i}+ \lambda_{2}^{*} \eta_{i0}+\lambda_{3}^{*} \eta_{i1}+\lambda_{4}^{*} \varpi+\varepsilon_{yi}^{*}$ ()

The parameters $\alpha_{0}^{*}$, $\alpha_{1}^{*}$, $\alpha_{2}^{*}$, $\alpha_{3}^{*}$, $\lambda_{1}^{*}, \lambda_{2}^{*}$,$\lambda_{3}^{*}{, \gamma}_{01}^{*}$ and $\gamma_{11}^{*}$ in the latent augmented model parameters in ()-() have a similar interpretation to the corresponding parameters in the correlated augmented model in (1)-(5). The four confounder parameters $\gamma_{02}^{*}$, $\gamma_{12}^{*}$, $\lambda_{4}^{*}$, and $\lambda_{5}^{*}$ quantify the biasing effects of the latent confounder on the model parameters. The coefficients $\gamma_{02}^{*}$ and $\gamma_{12}^{*}$ are the effects of the latent confounder on the latent intercept and slope controlling for the antecedent variable, respectively; $\lambda_{4}^{*}$ is the effect of the latent confounder controlling for the antecedent variable and latent intercept and slope; $\lambda_{5}^{*}$ is the effect of the latent confounder on the antecedent variable.

## Assumptions

Before deriving analytic results, we make the following assumptions. 1- because $x_{i}$ is not randomized, it can be potentially influenced by an omitted confounder. We assume that there no other sources of bias in the model: that is, 1- the functional forms in the mediation model are correctly specified; 2- there are no common methods effect would explain additional covariance/correlation between the residuals in the model.

For the correlated augmented model, we assume that the correlations between residuals, except for the residuals associated with the latent intercept and slope, model the correlations induced by an omitted confounder and not by the correlations induced by additional sources of bias (e.g., common methods effect). For the latent augmented model, we make the following assumptions: 1- we assume that ϖ include all the linear combinations of the omitted confounders. As such, when included in the model, ϖ does not influence the residuals at either Within or Between level. 2- we also assume constant variance for the Between residuals in the model. For the Within residuals, however, each time point can have a unique constant variance.

Next, we derive analytic results for the correlated augmented model and then present the results for the latent augmented model. In the end, we compare the results for two models to show equivalency between the corresponding parameters and to show confounder correlations/covariances in the correlated augmented model are a unique function of the model parameters, including the confounding parameters, in the latent augmented model.

## Correlated Augmented Model

We first derive expected values of the mediators and outcome variable as follows:

 () ()

 ()

We next derive covariances between model variables and residuals.

$\mathrm{cov}(x_{i},\zeta_{1i}^{**})=\mathrm{cov}(\varepsilon_{xi}^{**},\zeta_{1i}^{**})$ ()

The above result shows that the covariance between $x_{i}$ and $\zeta_{1i}^{**}$is the same as the one between the residual associated with $x_{i}$, $\varepsilon_{1i}^{**}$, and $\zeta_{1i}^{**}$.

$\mathrm{cov}\left(\eta_{i0},\eta_{i1}\right)=\gamma_{01}^{**}\gamma_{11}^{**} \sigma_{\varepsilon_{xi}^{*}}^{2}+\gamma_{01}^{**} \mathrm{cov}(\varepsilon_{xi}^{**}, \zeta_{1i}^{**})+\gamma_{11}^{**}\mathrm{cov}(\varepsilon_{xi}^{**},\zeta_{0i}^{**})+\mathrm{cov}(\zeta_{0i}^{**}, \zeta_{1i}^{**})$ ()

$\mathrm{cov}(\eta_{i0},x_{i})=\mathrm{cov}(\alpha_{0}^{**}+\gamma_{01}^{**} x_{i}+ \zeta_{0i}^{**},x_{i})=\gamma_{01}^{**}\sigma_{\varepsilon_{xi}^{**}}^{2}+\mathrm{cov}(\zeta_{0i}^{**},\varepsilon_{xi}^{**})$ ()

$\mathrm{cov}(\eta_{i1},x_{i})=\gamma_{11}^{**}\sigma_{\varepsilon_{xi}^{**}}^{2}+\mathrm{cov}(\zeta_{1i}^{**},\varepsilon_{xi}^{**})$ ()

$\begin{matrix} \mathrm{cov}(y_{i},x_{i})=\mathrm{cov}(\alpha_{2}^{**}+\kappa^{**} x_{i}+ \lambda_{1}^{**} \eta_{i0}+\lambda_{3}^{**} \eta_{i1}+\varepsilon_{yi}^{**},x_{i}) \\ =\left(\kappa^{**}+\lambda_{1}^{**}\gamma_{01}^{**}+\lambda_{2}^{**}\gamma_{11}^{**}\right)\sigma_{x}^{2}+\lambda_{1}^{**}\mathrm{cov}(\zeta_{0i}^{**},\varepsilon_{xi}^{**})+\lambda_{2}^{**}\mathrm{cov}(\zeta_{1i}^{**},\varepsilon_{xi}^{**})+\mathrm{cov}(x_{i},\varepsilon_{yi}^{**}) \end{matrix}$ ()

$\begin{matrix} \mathrm{cov}(\eta_{i0},y_{i})=\mathrm{cov}\left(\alpha_{0}^{**}+\gamma_{01}^{**} x_{i}+ \zeta_{0i}^{**}, \alpha_{2}^{**}+\kappa^{**} x_{i}+ \lambda_{1}^{**} \eta_{i0}+\lambda_{2}^{**} \eta_{i1}+\varepsilon_{yi}^{**}\right) \\ =\kappa^{**}\left(\gamma_{01}^{**}\kappa^{**}\sigma_{\varepsilon_{xi}^{**}}^{2}+\mathrm{cov}( \zeta_{0i}^{**},x_{i})\right) \\ +\lambda_{1}^{**} \left(\left(\gamma_{01}^{**}\right)^{2}\sigma_{\varepsilon_{xi}^{**}}^{2}+2\gamma_{01}^{**}\mathrm{cov}( \zeta_{0i}^{**},x_{i})+\sigma_{\zeta_{0i}^{**}}^{2}\right) \\ +\lambda_{2}^{**} \left(\gamma_{01}^{**}\gamma_{11}^{**}\sigma_{\varepsilon_{xi}^{**}}^{2}+\gamma_{01}^{**}\mathrm{cov}( \zeta_{1i}^{**},x_{i})+\gamma_{11}^{**}\mathrm{cov}( \zeta_{0i}^{**},x_{i})+\mathrm{cov}(\zeta_{0i}^{**},\zeta_{1i}^{**})\right) \\ +\gamma_{01}^{**}\mathrm{cov}(x_{i},\varepsilon_{yi}^{**})+\mathrm{cov}( \zeta_{0i}^{**},\varepsilon_{yi}^{**}) \end{matrix}$ ()

$\begin{matrix} \mathrm{cov}(\eta_{i1},y_{i})=\mathrm{cov}(\eta_{i1},\alpha_{2}^{**}+\kappa^{**} x_{i}+ \lambda_{1}^{**} \eta_{i0}+\lambda_{2}^{**} \eta_{i1}+\varepsilon_{yi}^{**}) \\ =\kappa^{**}\left(\gamma_{11}^{**}\sigma_{\varepsilon_{xi}^{**}}^{2}+\mathrm{cov}(\zeta_{1i}^{**},\varepsilon_{xi}^{**})\right) \\ +\lambda_{1}^{**}\left(\gamma_{01}^{**}\gamma_{11}^{**} \sigma_{\varepsilon_{xi}^{*}}^{2}+\gamma_{01}^{**} \mathrm{cov}(\varepsilon_{xi}^{**}, \zeta_{1i}^{**})+\gamma_{11}^{**}\mathrm{cov}(\varepsilon_{xi}^{**},\zeta_{0i}^{**})+\mathrm{cov}(\zeta_{0i}^{**}, \zeta_{1i}^{**})\right) \\ +\lambda_{2}^{**}\left(\left(\gamma_{11}^{**}\right)^{2}\sigma_{x}^{2}+\sigma_{\zeta_{1i}^{**}}^{2}+\gamma_{11}^{**}\mathrm{cov}(\varepsilon_{xi}^{**},\zeta_{1i}^{**})\right) \\ +\gamma_{11}^{**}\mathrm{cov}(\varepsilon_{xi}^{**},\varepsilon_{yi}^{**})+\mathrm{cov}(\zeta_{1i}^{**},\varepsilon_{yi}^{**}) \end{matrix}$ ()

## Latent Augmented Model

We first derive expected values of the mediators and outcome variable as follows:

 ()

 ()

 ()

Next, we derive covariance between the residuals and variables in the model.
$\mathrm{cov}(\eta_{i0},x_{i})=\gamma_{01}^{*}\sigma_{x}^{2}+\gamma_{02}^{*}\lambda_{4}^{*}\sigma_{\varpi}^{2}$ ()

$\mathrm{cov}(\eta_{i1},x_{i})=\gamma_{11}^{*}\sigma_{x}^{2}+\gamma_{12}^{*}\lambda_{4}^{*} \sigma_{\varpi}^{2}$ ()

$\begin{matrix} \mathrm{cov}(y_{i},x_{i})=\mathrm{cov}\left(\alpha_{2}^{*}+\kappa^{*} x_{i}+ \lambda_{1}^{*} \eta_{i0}+\lambda_{2}^{*} \eta_{i1}+\lambda_{3}^{*} \varpi+\varepsilon_{yi}^{*},x_{i}\right) \\ =\left(\kappa^{*}+\lambda_{1}^{*}\gamma_{01}^{*}+\lambda_{2}^{*}\gamma_{11}^{*}\right)\sigma_{x}^{2}+\lambda_{1}^{*}\gamma_{02}^{*}\lambda_{4}^{*}\sigma_{\varpi}^{2}+\lambda_{2}^{*}\gamma_{12}^{*}\lambda_{4}^{*}\sigma_{\varpi}^{2} +\lambda_{3}^{*} \lambda_{4}^{*}\sigma_{\varpi}^{2} \end{matrix}$ ()

$\begin{matrix} \mathrm{cov}(\eta_{i0},\eta_{i1})=\mathrm{cov}(\alpha_{0}^{*}+\gamma_{01}^{*}x_{i}+\gamma_{02}^{*}\varpi+\zeta_{0i}^{*},\alpha_{1}^{*}+\gamma_{11}^{*}x_{i}+\gamma_{12}^{*} \varpi+\zeta_{1i}^{*}) \\ =\gamma_{01}^{*}\gamma_{11}^{*}\sigma_{x}^{2}+\left(\gamma_{01}^{*}\gamma_{12}^{*} +\gamma_{02}^{*}\gamma_{11}^{*}\right)\lambda_{4}^{*}\sigma_{\varpi}^{2}+\gamma_{02}^{*}\gamma_{12}^{*}\sigma_{\varpi}^{2}+\mathrm{cov}(\zeta_{0i}^{*},\zeta_{1i}^{*}) \end{matrix}$ ()

Note that the result below holds because we assumed that the ϖ accounts for all confounder correlations in the model, and thus there is no residual correlation left unmodeled.

$\mathrm{cov}(\eta_{i1},\varepsilon_{yi}^{*})=0$ ()

We use the result () in deriving the covariance between $\eta_{i0}$ and $y_{i}$:

$\begin{matrix} \mathrm{cov}(\eta_{i0},y_{i})=\mathrm{cov}(\alpha_{2}^{*}+\kappa^{*} x_{i}+ \lambda_{1}^{*} \eta_{i0}+\lambda_{2}^{*} \eta_{i1}+\lambda_{3}^{*} \varpi+\varepsilon_{yi}^{*},\eta_{i0}) \\ =\kappa^{*}\left[\gamma_{01}^{*}\sigma_{x}^{2}+\gamma_{02}^{*}\lambda_{4}^{*}\sigma_{\varpi}^{2}\right] \\ +\lambda_{1}^{*} \left(\left(\gamma_{01}^{*}\right)^{2}\sigma_{x}^{2}+\left(\gamma_{02}^{*}\right)^{2}\sigma_{\varpi}^{2}+2\gamma_{01}^{*}\gamma_{02}^{*}\lambda_{4}^{*}\sigma_{\varpi}^{2}+\sigma_{\zeta_{0i}^{*}}^{2}\right) \\ +\lambda_{2}^{*}\left[\gamma_{01}^{*} \gamma_{11}^{*}\sigma_{x}^{2}+\gamma_{01}^{*}\lambda_{4}^{*}\gamma_{12}^{*}\sigma_{\varpi}^{2}+\gamma_{11}^{*}\lambda_{4}^{*} \gamma_{02}^{*}\sigma_{\varpi}^{2}+\gamma_{02}^{*}\gamma_{12}^{*}\sigma_{\varpi}^{2}+\mathrm{cov}(\zeta_{0i}^{*}, \zeta_{1i}^{*})\right] \\ +\gamma_{01}^{*}\lambda_{3}^{*} \lambda_{4}^{*}\sigma_{\varpi}^{2} +\lambda_{3}^{*}\gamma_{02}^{*}\sigma_{\varpi}^{2} \end{matrix}$ ()

$\begin{matrix} \mathrm{cov}(\eta_{i1},y_{i})=\mathrm{cov}(\eta_{i1},\alpha_{2}^{*}+ \kappa^{*}x_{i}+ \lambda_{1}^{*} \eta_{i0}+\lambda_{2}^{*} \eta_{i1}+\lambda_{3}^{*} \varpi+\varepsilon_{yi}^{*}) \\ =\kappa^{*}\left(\gamma_{11}^{*}\sigma_{x}^{2}+\gamma_{12}^{*}\lambda_{4}^{*} \sigma_{\varpi}^{2}\right) \\ +\lambda_{1}^{*}\left(\gamma_{01}^{*}\gamma_{11}^{*}\sigma_{x}^{2}+\left(\gamma_{01}^{*}\gamma_{12}^{*} +\gamma_{02}^{*}\gamma_{11}^{*}\right)\lambda_{4}^{*}\sigma_{\varpi}^{2}+\gamma_{02}^{*}\gamma_{12}^{*}\sigma_{\varpi}^{2}+\mathrm{cov}(\zeta_{0i}^{*},\zeta_{1i}^{*})\right) \\ +\lambda_{2}^{*}\left(\left(\gamma_{11}^{*}\right)^{2}\sigma_{x}^{2}+\left(\gamma_{12}^{*}\right)^{2}\sigma_{\varpi}^{2}+2\gamma_{11}^{*}\gamma_{12}^{*}\lambda_{4}^{*}\sigma_{\varpi}^{2}+\sigma_{\zeta_{1i}^{*}}^{2}\right) \\ +\gamma_{11}^{*}\lambda_{3}^{*}\lambda_{4}^{*}\sigma_{\varpi}^{2}+\lambda_{3}^{*}\gamma_{12}^{*}\sigma_{\varpi}^{2} \end{matrix}$ ()

## Results

Comparing () and () corresponding terms, we conclude that

$\begin{matrix} \alpha_{0}^{*}=\alpha_{0}^{**} \\ \gamma_{01}^{*}=\gamma_{01}^{**} \end{matrix}$ ()

Comparing () and (),

$\begin{matrix} \alpha_{1}^{*}=\alpha_{1}^{**} \\ \gamma_{11}^{*}=\gamma_{11}^{**} \end{matrix}$ ()

Comparing () and (), we have

$\begin{matrix} \lambda_{1}^{*}=\lambda_{1}^{**} \\ \lambda_{2}^{*}=\lambda_{2}^{**} \\ \lambda_{3}^{*}=\lambda_{3}^{**} \end{matrix}$ ()

From (), (), and (), we conclude that direct and indirect effects from two models are equivalent:

$\begin{matrix} \gamma_{01}^{*}\lambda_{2}^{*}=\gamma_{01}^{**}\lambda_{2}^{**} \\ \gamma_{11}^{*}\lambda_{3}^{*}=\gamma_{11}^{**}\lambda_{3}^{**} \\ \lambda_{1}^{*}=\lambda_{1}^{**} \end{matrix}$ ()

Comparing () with (), and () with (), we have

$\mathrm{cov}(\varepsilon_{xi}^{**},\zeta_{0i}^{**})=\rho_{2}\sigma_{\varepsilon_{xi}^{**}}\sigma_{\zeta_{0i}^{**}}=\gamma_{02}^{*}\lambda_{5}^{*}\sigma_{\varpi}^{2}$ ()

$\mathrm{cov}(\varepsilon_{xi}^{**},\zeta_{1i}^{**})=\rho_{3}\sigma_{\varepsilon_{xi}^{**}}\sigma_{\zeta_{1i}^{**}}=\gamma_{12}^{*}\lambda_{4}^{*}\sigma_{\varpi}^{2}$ ()

Comparing the corresponding terms in () and (), we have:

$\mathrm{cov}(\varepsilon_{xi}^{**},\varepsilon_{yi}^{**})=\rho_{1}\sigma_{\varepsilon_{x}^{**}}\sigma_{\varepsilon_{y}^{**}}=\lambda_{4}^{*} \lambda_{5}^{*}\sigma_{\varpi}^{2}$ ()

Comparing () and (), we have the following relationships:

$\mathrm{cov}(\zeta_{0i}^{**},\varepsilon_{yi}^{**})=\rho_{4}\sigma_{\zeta_{0i}^{**}}\sigma_{\varepsilon_{yi}^{**}}=\lambda_{3}^{*}\gamma_{02}^{*}\sigma_{\varpi}^{2}$ ()

$\mathrm{cov}(\zeta_{0i}^{**},\zeta_{1i}^{**})=\rho_{6}\sigma_{\zeta_{0i}^{**}}\sigma_{\zeta_{1i}^{**}}=\gamma_{02}^{*}\gamma_{12}^{*}\sigma_{\varpi}^{2}+\mathrm{cov}(\zeta_{0i}^{*}, \zeta_{1i}^{*})$ ()

Comparing (A19) and (A37), we have

$\mathrm{cov}(\zeta_{1i}^{**},\varepsilon_{yi}^{**})=\rho_{5}\sigma_{\zeta_{1i}^{**}}\sigma_{\varepsilon_{yi}^{**}}=\lambda_{3}^{*}\gamma_{12}^{*}\sigma_{\varpi}^{2}$.

## Summary

Below, we show a summary of the key analytic results that exhibit equivalence between the latent augmented and the correlated augmented models.

$\gamma_{11}^{*}=\gamma_{11}^{**}$ ()

$\lambda_{1}^{*}=\lambda_{1}^{**}$ ()

$\lambda_{2}^{*}=\lambda_{2}^{**}$ ()

$\lambda_{3}^{*}=\lambda_{3}^{**}$ ()

From () to (), we conclude that the indirect effects from the two models are equivalent:

$\gamma_{01}^{*}\lambda_{2}^{*}=\gamma_{01}^{**}\lambda_{2}^{**}$ ()

$\gamma_{11}^{*}\lambda_{3}^{*}=\gamma_{11}^{**}\lambda_{3}^{**}$ ()

The results () and () show that, if we use either the latent or the correlated augmented model, we estimate the same quantities for the indirect effects. An important implication is that the correlated augmented model exhibits the same results as the ones from the latent augmented model, where the latent proxy variable ϖ explicitly models a linear combination of effects of confounders. Thus, if we use the correlated augmented model, we will estimate the same quantities for the indirect effects as the ones in the latent augmented model.

$\mathrm{cov}(\varepsilon_{xi}^{**},\zeta_{0i}^{**})=\gamma_{02}^{*}\lambda_{4}^{*}\sigma_{\varpi}^{2}$ ()

$\mathrm{cov}(\varepsilon_{xi}^{**},\zeta_{1i}^{**})=\gamma_{12}^{*}\lambda_{4}^{*}\sigma_{\varpi}^{2}$ ()

$\mathrm{cov}(\varepsilon_{xi}^{**},\varepsilon_{yi}^{**})=\lambda_{3}^{*} \lambda_{4}^{*}\sigma_{\varpi}^{2}$ ()

$\mathrm{cov}(\zeta_{0i}^{**},\varepsilon_{yi}^{**})=\lambda_{3}^{*}\gamma_{02}^{*}\sigma_{\varpi}^{2}$ ()

$\mathrm{cov}(\zeta_{1i}^{**},\varepsilon_{yi}^{**})=\lambda_{3}^{*}\gamma_{12}^{*}\sigma_{\varpi}^{2}$ ()

$\mathrm{cov}(\zeta_{0i}^{**},\zeta_{1i}^{**})=\gamma_{02}^{*}\gamma_{12}^{*}\sigma_{\varpi}^{2}+\mathrm{cov}(\zeta_{0i}^{*},\zeta_{1i}^{*})$ ()

# R Code to Run CAMSA in Nonrandomized Longitudinal Mediation Analysis

# Reading Data

df <- read_spss("../data/COMBINE pain and affect factors_opioids.sav")

df <- df %>% rename_all(tolower)

df_sub <- df %>% dplyr::select(pain16, m16pdd, blpdd,
affect16, affect26, affect52, affect68,
gender, married, employ, income, minority,
adstot, alcdepnd, readines, confid0)

## Standardize
df_sub1 <- df_sub %>% mutate_each(list(scale), starts_with("affect"), m16pdd, blpdd, pain16, employ, income,adstot, alcdepnd, readines, confid0)

## Warning: `mutate_each_()` was deprecated in dplyr 0.7.0.
## Please use `across()` instead.
## This warning is displayed once every 8 hours.
## Call `lifecycle::last_lifecycle_warnings()` to see where this warning was generated.

# Correlated Augmented Model

Below is the initial correlated augmented model. All the confounder correlations are fixed to zero. Later, this model will be modified to conduct CAMSA using combinations of correlation confounders.

m2 <- '
# intercept
i =~ 1*affect16 + 1*affect26 + 1*affect52 + 1*affect68
# slope
s =~ 0*affect16 + 2.6*affect26 + 3.6*affect52 + 5.2*affect68

f_pain =~ 1*pain16 # convert the antecedent variable into exogenous variable
pain16 ~~ 0*pain16; # fix residual variance for observed antecedent variable at 0 to make the model identified

# regression
i ~ a_i*f_pain
s ~ a_s*f_pain
i + s + f_pain ~ gender +married+ employ+ income+ minority+ adstot+ alcdepnd+ readines+ confid0;

m16pdd ~ blpdd + f_pain + gender +married+ employ+ income+ minority+ adstot+ alcdepnd+ readines+ confid0;

m16pdd ~ b_i* i + b_s*s;

i ~~ cov_is*s;
i ~~ s2_i* i;
s ~~ s2_s* s;

m16pdd ~~ s2_y*m16pdd
f_pain ~~ s2_x*f_pain

f_pain ~~ cov_xy*m16pdd # covariance between x and y
f_pain ~~ cov_xm1*i #covariance between x and m1
f_pain ~~ cov_xm2*s #covariance between x and m2
i ~~ cov_m1y*m16pdd #covariance between m1 and y
s ~~ cov_m2y*m16pdd #covariance between m2 and y

#Indirect effects

 ind_i := a_i*b_i;
 ind_s := a_s*b_s;

# Convert covariances to correlations

 r_is := cov_is/sqrt(s2_i*s2_s)
 rho_xy := cov_xy/sqrt(s2_x*s2_y)
 rho_xm1 := cov_xm1/sqrt(s2_x*s2_i)
 rho_xm2 := cov_xm2/sqrt(s2_x*s2_s)
 rho_m1y := cov_m1y/sqrt(s2_i*s2_y)
 rho_m2y := cov_m2y/sqrt(s2_s*s2_y)

 # constraints

 cov_xy == 0
 cov_xm1 == 0
 cov_xm2 == 0
 cov_m1y == 0
 cov_m2y == 0
'

fit2 <- growth(m2,
 data = df_sub1,
 missing = "fiml",
 fixed.x = FALSE)

res2 <- lavaan::parameterEstimates(fit2) %>% dplyr::filter(grepl("ind", lhs)) %>% dplyr::select(lhs,est,se, ci.lower,ci.upper)

res2

## lhs est se ci.lower ci.upper
## 1 ind_i 0.096 0.014 0.069 0.124
## 2 ind_s -0.013 0.012 -0.037 0.012

# CAMSA

## Toeplitz Method

Below, we conduct CAMSA. The code below replaces the confounder covariances in the initial correlated augmented model with the ones we specified using the Toeplitz method.

fit2_tbl <- parTable(fit2)
tbl1 <- fit2_tbl %>% dplyr::filter(op != '==')
tbl2 <- fit2_tbl %>% dplyr::filter(op == '==')
var_vec <- coef(fit2)[c('s2_x', 's2_i', 's2_s', 's2_y')]
sd_vec <- sqrt(var_vec)

cl <- makeCluster(detectCores() - 1)
registerDoParallel(cl)
res <- foreach(
 i = seq(.05, .5, by = .001),
 .errorhandling = "stop",
 .combine = "rbind",
 .packages = c("lavaan", "dplyr", "base", "tibble")
) %dopar% {
 rho_mat <- toeplitz(first_row(i))
 rownames(rho_mat) <-
 colnames(rho_mat) <- c('x', 'm1', 'm2', 'y')
 cov_mat <-
 lavaan::cor2cov(rho_mat, sd_vec, names = c('x', 'm1', 'm2', 'y'))
 cov_xy <- cov_mat['x', 'y']
 cov_xm1 <- cov_mat['x', 'm1']
 cov_xm2 <- cov_mat['x', 'm2']
 cov_m1m2 <- cov_mat['m1', 'm2']
 cov_m1y <- cov_mat['m1', 'y']
 cov_m2y <- cov_mat['m2', 'y']

 rho_xy <- rho_mat['x', 'y']
 rho_xm1 <- rho_mat['x', 'm1']
 rho_xm2 <- rho_mat['x', 'm2']
 rho_m1m2 <- rho_mat['m1', 'm2']
 rho_m1y <- rho_mat['m1', 'y']
 rho_m2y <- rho_mat['m2', 'y']

 r_vec <- c(rho_xy, rho_xm1, rho_xm2, rho_m1m2, rho_m1y, rho_m2y)
 r_mat <- rbind(r_vec, r_vec)
 colnames(r_mat) <-
 c("rho_xy",
 "rho_xm1",
 "rho_xm2",
 "rho_m1m2",
 "rho_m1y",
 "rho_m2y")
 r_mat <- tibble::as_tibble(r_mat)

 tbl_aug <- tbl2 %>%
 dplyr::mutate(
 rhs = replace(rhs, lhs == 'cov_xy', cov_xy),
 rhs = replace(rhs, lhs == 'cov_xm1', cov_xm1),
 rhs = replace(rhs, lhs == 'cov_xm2', cov_xm2),
 rhs = replace(rhs, lhs == 'cov_m1y', cov_m1y),
 rhs = replace(rhs, lhs == 'cov_m2y', cov_m2y)
 )

 tbl_new <- dplyr::bind_rows(tbl1, tbl_aug)
 fit2_new <- tryCatch(
 lavaan::growth(
 tbl_new,
 data = df_sub1,
 missing = "fiml",
 fixed.x = FALSE
 ),
 warning = function(w)
 return(NULL),
 error = function(e)
 return(NULL)
 )
 res1 <- if (!is.null(fit2_new))
 lavaan::parameterEstimates(fit2_new) %>% dplyr::filter(grepl("ind", lhs)) %>% dplyr::select(lhs, est, ci.lower, ci.upper)
 else {
 mat <- matrix(rep(NA, 8), nrow = 2)
 colnames(mat) <- c("lhs", "est", "ci.lower", "ci.upper")
 tibble::as_tibble(mat)
 }
 res1 <- tibble::as_tibble(res1)
 res2 <- dplyr::bind_cols(r_mat, res1)
 res2
}

stopCluster(cl)
knitr::kable(res)
writexl::write_xlsx(res,"sensitivity_table1.xlsx")

# Near PD Method

Below, we conduct CAMSA using the confounder correlations with the Near-PD method.

fit2_tbl <- parTable(fit2)
tbl1 <- fit2_tbl %>% dplyr::filter(op != '==')
tbl2 <- fit2_tbl %>% dplyr::filter(op == '==')
var_vec <- coef(fit2)[c('s2_x', 's2_i', 's2_s', 's2_y')]
sd_vec <- sqrt(var_vec)

r_vec <- seq(0, .5, by = .05)

r_df <-
 setDT(tidyr::expand_grid(
 rho_x = 1,
 rho_xm1 = r_vec,
 rho_m1 = 1,
 rho_xm2 = r_vec,
 rho_m1m2 = r_vec,
 rho_m2 = 1,
 rho_xy = r_vec,
 rho_m1y = r_vec,
 rho_m2y = r_vec,
 rho_y = 1
 ))


cl <- makeCluster(detectCores() - 1)
registerDoParallel(cl)
res <- foreach(
 i = iter(r_df, by='row'),
 .errorhandling = "stop",
 .combine = "bind_rows",
 .packages = c("lavaan", "dplyr", "base", "tibble", "Matrix"),
 .inorder = FALSE,
 .verbose = FALSE
) %dopar% {

 rho_mat <- lav_matrix_vechr_reverse(as.numeric(i))

 if (det(rho_mat) < 0)
 rho_mat <- Matrix::nearPD(
 rho_mat,
 corr = TRUE,
 keepDiag = TRUE,
 ensureSymmetry = TRUE
 )$mat

 rownames(rho_mat) <-
 colnames(rho_mat) <- c('x', 'm1', 'm2', 'y')
 #cov_mat <-
 # lavaan::cor2cov(rho_mat, sd_vec, names = c('x', 'm1', 'm2', 'y'))
 D <- solve(diag(sd_vec))
 cov_mat <- D %*% rho_mat %*% D
 rownames(cov_mat) <-
 colnames(cov_mat) <- c('x', 'm1', 'm2', 'y')
 cov_xy <- cov_mat['x', 'y']
 cov_xm1 <- cov_mat['x', 'm1']
 cov_xm2 <- cov_mat['x', 'm2']
 cov_m1m2 <- cov_mat['m1', 'm2']
 cov_m1y <- cov_mat['m1', 'y']
 cov_m2y <- cov_mat['m2', 'y']

 rho_xy <- rho_mat['x', 'y']
 rho_xm1 <- rho_mat['x', 'm1']
 rho_xm2 <- rho_mat['x', 'm2']
 rho_m1m2 <- rho_mat['m1', 'm2']
 rho_m1y <- rho_mat['m1', 'y']
 rho_m2y <- rho_mat['m2', 'y']

 r_vec <- c(rho_xy, rho_xm1, rho_xm2, rho_m1m2, rho_m1y, rho_m2y)
 r_mat <- rbind(r_vec, r_vec)
 colnames(r_mat) <-
 c("rho_xy",
 "rho_xm1",
 "rho_xm2",
 "rho_m1m2",
 "rho_m1y",
 "rho_m2y")
 r_mat <- tibble::as_tibble(r_mat)

 tbl_aug <- tbl2 %>%
 dplyr::mutate(
 rhs = replace(rhs, lhs == 'cov_xy', cov_xy),
 rhs = replace(rhs, lhs == 'cov_xm1', cov_xm1),
 rhs = replace(rhs, lhs == 'cov_xm2', cov_xm2),
 rhs = replace(rhs, lhs == 'cov_m1y', cov_m1y),
 rhs = replace(rhs, lhs == 'cov_m2y', cov_m2y)
 )

 tbl_new <- dplyr::bind_rows(tbl1, tbl_aug)
 fit2_new <- tryCatch(
 lavaan::growth(
 tbl_new,
 data = df_sub1,
 missing = "fiml",
 fixed.x = FALSE
 ),
 warning = function(w)
 return(NULL),
 error = function(e)
 return(NULL)
 )
 res1 <- if (!is.null(fit2_new))
 lavaan::parameterEstimates(fit2_new) %>% dplyr::filter(grepl("ind", lhs)) %>% dplyr::select(lhs, est, ci.lower, ci.upper)
 else {
 mat <- matrix(rep(NA, 8), nrow = 2)
 colnames(mat) <- c("lhs", "est", "ci.lower", "ci.upper")
 tibble::as_tibble(mat)
 }
 res1 <- tibble::as_tibble(res1)
 res2 <- dplyr::bind_cols(r_mat, res1)
 res2
}

stopCluster(cl)
writexl::write_xlsx(res,"sensitivity_table_1NPD.xlsx")
